# Enhancement of Ultraviolet Light Resistance of Colorless and Transparent Semi-Alicyclic Polyimide Nanocomposite Films via the Incorporation of Hindered Amine Light Stabilizers for Potential Applications in Flexible Optoelectronics

**DOI:** 10.3390/polym14061091

**Published:** 2022-03-09

**Authors:** Xin-Ying Wei, Zhi-Bin He, Shun-Qi Yuan, Hao Wu, Xin-Xin Zhi, Yan Zhang, Shu-Jing Chen, Jin-Gang Liu

**Affiliations:** 1 Beijing Key Laboratory of Materials Utilization of Nonmetallic Minerals and Solid Wastes, National Laboratory of Mineral Materials, School of Materials Science and Technology, China University of Geosciences, Beijing 100083, China; 2003200022@cugb.edu.cn (X.-Y.W.); 2003200021@cugb.edu.cn (H.W.); 3003200015@cugb.edu.cn (X.-X.Z.); 3003200016@cugb.edu.cn (Y.Z.); 2 RAYITEK Hi-Tech Film Co., Ltd., Shenzhen 518105, China; zb.he@rayitek.cn (Z.-B.H.); sq.yuan@rayitek.cn (S.-Q.Y.)

**Keywords:** colorless polyimide, optical property, UV exposure, hindered amine light stabilizers

## Abstract

Optically transparent polymer films with excellent thermal and ultraviolet (UV) resistance have been highly desired in advanced optoelectronic fields, such as flexible substrates for photovoltaic devices. Colorless and transparent polyimide (CPI) films simultaneously possess the good thermal stability and optical transparency. However, conventional CPI films usually suffered from the UV exposure and have to face the deterioration of optical properties during the long-term service in UV environments. In the current work, the commercially available hindered amine light stabilizers (HALS) were tried to be incorporated into the semi-alicyclic CPI matrix with the aim of enhancing the UV exposure stability. For this target, a CPI-0 film was first prepared from hydrogenated pyromellitic dianhydride (HPMDA) and 2,2′-dimethylbenzidine (DMBZ) via a one-step polycondensation procedure. Then, the commercially available HALS were incorporated into the CPI-0 (HPMDA-DMBZ) film matrix to afford four series of CPI/HALS composite films. Experimental results indicated that the Tinuvin^®^ 791 HALS showed the best miscibility with the CPI-0 film matrix and the derived CPI-D series of composite films exhibited the best optical transmittances. The CPI-D nanocomposite films showed apparently enhanced UV exposure stability via incorporation of the 791 additives. For the pristine CPI-0 film, after the UV exposure for 6 h, the optical properties, including the transmittance at the wavelength of 350 nm (*T*_350_), lightness (L*), yellow indices (b*), and haze obviously deteriorated with the *T*_350_ values from 55.7% to 17.5%, the L* values from 95.12 to 91.38, the b* values from 3.38 to 21.95, and the haze values from 1.46% to 9.33%. However, for the CPI-D-10 film (791: CPI-0 = 1.0 wt%, weight percent), the optical parameters were highly maintained with the *T*_350_ values from 61.4% to 53.8%, the L* values from 95.46 to 95.36, the b* values from 1.84 to 1.51, and the haze values from 0.69% to 3.34% under the same UV aging conditions.

## 1. Introduction

Colorless and transparent polyimide (CPI) films have been well known for the excellent combined thermal and optical properties for many years in advanced optical and optoelectronic applications [1,2,3]. In many practical applications, CPI films have to be 1qconfronted with the long-term ultraviolet-visible (UV) light exposure, such as the fluorinated CPI thermal control films for the space aircrafts have to endure the large doses of ultraviolet (UV) and vacuum ultraviolet (VUV) irradiation during the servicing life, and the CPI substrates for flexible photovoltaic solar cells will be irradiated by sunlight in the whole life. Generally speaking, due to the intrinsic organic molecular chain composition and the covalent bond structural features, the molecular chains and chemical bonds in organic polymers might be broke after long-term irradiation of UV and visible lights, especially the high-energy UV-A (320–400 nm), UV-B (320–400 nm), and UV-C (100–280 nm) exposure [4,5,6,7,8,9]. The great deterioration in the optical, mechanical, and dielectric properties of the polymers might be caused. The same is true for the polyimide (PI) films. For the PI films, the carbonyl groups in the imide ring in the molecular chains will absorb UV energy, causing the ring-opening degradation of the imide rings. The photodegradation products might be further decomposed into volatile substances in the presence of oxygen, resulting in the weight loss and thickness reduction in the PI films. Therefore, the photodegradation and photo-stabilization issues have been becoming one of the most important research areas for PI films. Investigating the photodegradation mechanism of PI films, especially the CPI films, is of important theoretical and practical values to guarantee the reliabilities for long lasting services.

In 1990s, a series of pioneering work on the investigation of photo-degradation of PI films, especially the fluorinated PI films, were carried out in Hoyle’s group via spectral, viscometric, chromatographic, and weight loss methodologies [10,11,12,13,14,15]. The experimental results based on the systematic analysis for the photodegradation behaviors of various PI films demonstrated that the photodegradation behavior of PI films is closely related to the electronic properties of the substituents in their molecular structure. The presence of electron-withdrawing or electron-donating groups has quite different effects on the photodegradation behaviors of the PI films. The significantly different photo-stability between PI films with different structural features is closely related to the charge transfer (CT) interactions in the molecular chains, or the formation of charge transfer complex (CTC) [16]. The more stable the CTC is, the more difficult it is to release the high reactive species during light irradiation, the better the photo-stability of the PI films, and vice versa, PI films with weak CT interactions or poor ability to form CTC might possess relatively poor photo-stability. In the investigation, the fluorinated PI films derived from 4,4′-(hexafluoroisopropylidene)diphthalic anhydride (6FDA) and aromatic diamines showed obviously inferior UV exposure stability to the analogous PI films derived from other non-fluorinated dianhydrides, such as pyromellitic dianhydride (PMDA), 3,3′,4,4′-benzophenonetetracarboxylic dianhydride (BTDA), and 4,4′-oxydiphthalic dianhydride (ODPA). Thus, it could be expected that the CPI films, either the fluoro-containing systems or the alicyclic systems with the intrinsic low CT interactions in the molecular structures, might be more easily to be eroded in long-term UV irradiation [17,18]. Very recently, Mai et al. reported the photodegradation of a semi-alicyclic CPI film derived from 1,2,3,4-cyclobutanetetracarboxylic dianhydride (CBDA) and a bio-derived diamine [19]. It was found that, after several hundred hours of UV irradiation at 550 W/m^2^ and 83 °C, the photodegradation of the CPI film took place at the position of cyclobutane, imide, and aromatic groups. The mechanical and optical properties of the CPI film apparently deteriorated after the UV irradiation. Thus, with the rapid development of energy and information industries for the increasing demands of high-performance CPI films, the photo-stability research of CPI films has been given increasing attention.

Considering the very limited effects on improving the UV irradiation resistance of CPI films via mere structural modification, the current research has mainly been focused on the combination of the CPI film matrix with specific light sensitizers. Various light stabilizers, such as UV absorbers, peroxide decomposition agents, and free radical quenchers, can be applied to the photo-stability of polymer films [20,21]. However, for CPI films, the suitable light stabilizers should meet several property requirements for practical applications, including the good miscibility with the CPI film matrix so as to maintain the intrinsic optical transparency and color parameters (yellow index, haze, et al. of the pristine CPI matrix), good solubility in the good solvents for CPI film production so as to avoid the aggregation of the light stabilizers and achieve the homogeneous blending; colorless or low color, and high thermal stability with the initial decomposition temperatures higher than the processing temperature for the CPI film (≥280 °C). According to these criteria, it is quite challenging for seeking out the suitable light stabilizers for CPI films. Kim and coworkers recently reported the applications of the benzotriazole derivatives as the UV absorbers for semi-alicyclic CPI film [22]. Hydroxyphenyl benzotriazole is one of the excited state intra-molecular proton transfer UV absorbers, which emits UV energy absorbed by the molecule through keto-enol tautomerization between proton donor and acceptor pairs in the molecule, resulting in nonradiative properties in the visible region. Such non-radiative properties can relax UV energy without hindering the optical property of the polymer. The highly crystalline feature and the strong hydrogen bonding interactions endowed the benzotriazole derivatives based on Tinuvin^®^ 327 monomer good thermal stability; however, they decreased the solubility in the good solvent of *N*-methyl-2-pyrrolidinone (NMP) (<0.5 wt%). Nevertheless, the CPI composite films showed obviously enhanced UV resistance during the Xenon lamp irradiation in the wavelength of 300–800 nm at 250–765 W/m^2^ for 40 h.

In the current work, another type of light stabilizer, hindered amine light stabilizers (HALS), was attempted to incorporate into the semi-alicyclic CPI films so as to improve the UV stability. Different from the benzotriazole UV absorbers, the HALS photo-stabilizer can achieve the photo-stabilization of the polymers by capturing the free radicals produced during the photo-oxidation and degradation, and decompose the hydrogen peroxide and quenching the excited state energy [23,24,25,26,27,28,29,30]. This operation mechanism is quite suitable for the photo-stabilization of CPI films according to the photodegradation mechanism of the polymers. However, to the best of our knowledge, few works have been reported in the literature in this field. Thus, the practicability of the HALS in the photo-stabilization of CPI films and effects of the incorporation of HALS on the thermal and optical properties of the derived CPI composite films were studied in detail in the current work.

## 2. Materials and Methods

### 2.1. Materials

1,2,4,5-Cyclohexanetetracarboxylic acid dianhydride (CHDA) (melting point: 303.0 °C) or hydrogenated pyromellitic dianhydride (HPMDA) was purchased from Weihai Newera Kesense New Materials Co., Ltd. (Weihai, China) and dried in vacuo overnight at 180 °C before use. 2,2′-Dimethylbenzidine (DMBZ) was purchased from Tokyo Chemical Industry Co., Ltd. (Tokyo, Japan) and used directly. Four commercially available hindered amine light sensitizers (HALSs), including Chimassorb^®^ 2020, Chimassorb^®^ 944, Tinuvin^®^ 791, and Tinuvin^®^ 783, were purchased from BASF East Asia (Hong Kong, China) and used as received. The ultra-dry γ-butyrolactone (GBL) and *N,N*-dimethylacetamide (DMAc) were purchased from Beijing Innochem Science & Technology Co., Ltd. (Beijing, China). The other reagent-grade chemicals were all purchased from Sinopharm Chemical Reagent Co., Ltd. (Shanghai, China) and were used as received.

### 2.2. Measurements

Number average molecular weight (*M_n_*) and weight average molecular weight (*M_w_*) of the CPI-0 (HPMDA-DMBZ) resin were measured using a Shimadzu gel permeation chromatography (GPC) system (Kyoto, Japan). Fourier transform infrared (FTIR) spectra were measured on a Shimadzu Iraffinity-1S FT-IR spectrometer (Kyoto, Japan) from 4000 to 400 cm^−^^1^. The optical transmittances of the CPI films were determined by a Hitachi UH5700 Ultraviolet-visible (UV-Vis) spectrophotometer (Tokyo, Japan). Wide-angle X-ray diffraction (XRD) was obtained on a Rigaku D/max-2500 X-ray diffractometer (Tokyo, Japan) using Cu-Kα1 radiation, 2*θ* ranging from 10° to 60°. The full-width at half-maximum (FWHM) values of the HALS and CPI samples were calculated using Scherrer’s equation. The color parameters of the CPI films at a thickness of 25 µm were measured by an X-rite color i7 spectrophotometer (Grand Rapids, MI, USA) and were calculated according to a CIE (International Commission on Illumination) Lab equation. L* is the lightness, where 100 means white and 0 implies black. a*: positive value means red, negative value indicates green; b*: positive value means yellow, negative value indicates blue. Thermogravimetric analysis (TGA) was performed using a TA-Q50 thermal analysis system (New Castle, DE, USA) at a heating rate of 20 °C/min under nitrogen ranged from 25 °C to 760 °C. Differential scanning calorimetry (DSC) was conducted on the PI films using a TA-Q100 thermal analysis system (New Castle, DE, USA) in standard aluminum crucible at a heating rate of 10 °C/min in nitrogen. Thermal mechanical analyzer (TMA) was carried out with a NETZSCH TMA402F3 thermal analysis system (Selb, Germany) in nitrogen at a heating rate of 10 °C/min. The coefficients of linear thermal expansion (CTE) of the films in the range of 50–250 °C were recorded.

**Figure 1:**
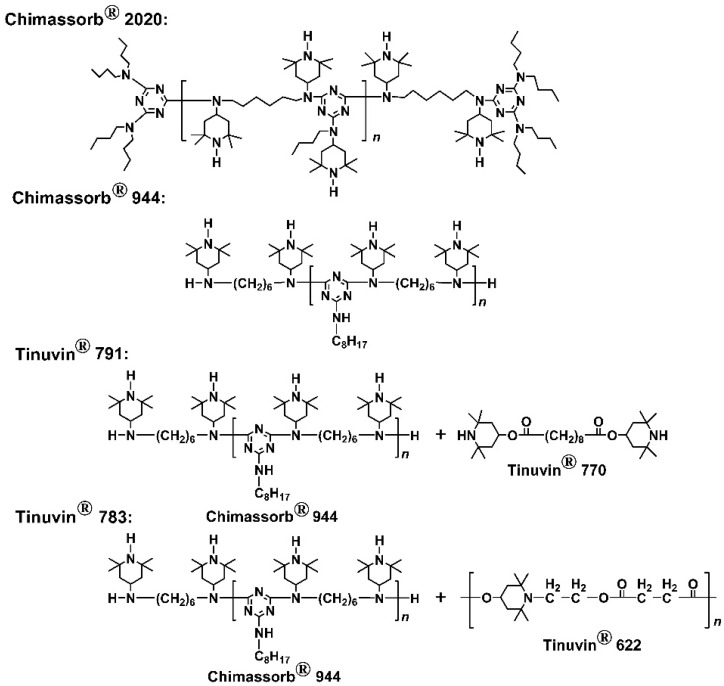
Commercially available hindered amine light sensitizers (HALS) used for the preparation of CPI/HALS nanocomposite films (Source: supplier).

The solubility of four commercially available HALS, including Chimassorb^®^ 2020, Chimassorb^®^ 944, Tinuvin^®^ 791, and Tinuvin^®^ 783, whose typical chemical structures are shown in Figure 1 in the good solvent of DMAc for the CPI-0 resin was evaluated by mixing 1.0 g of the HALS and 5.0 g of DMAc (16.7 wt% solid content) in a 10-mL glass bottle. The mixture was electromagnetically stirred at room temperature for 24 h. The solubility was visually determined.

Solubility of the CPI-0 resin in the tested solvents was investigated by mixing 1.0 g of the CPI resin and 9.0 g of the solvent tested (10.0 wt% solid content), and then stirred for 24 h at room temperature. The solubility was also determined visually as three grades: completely soluble (++), partially soluble (+), and insoluble (−).

**Figure 2:**
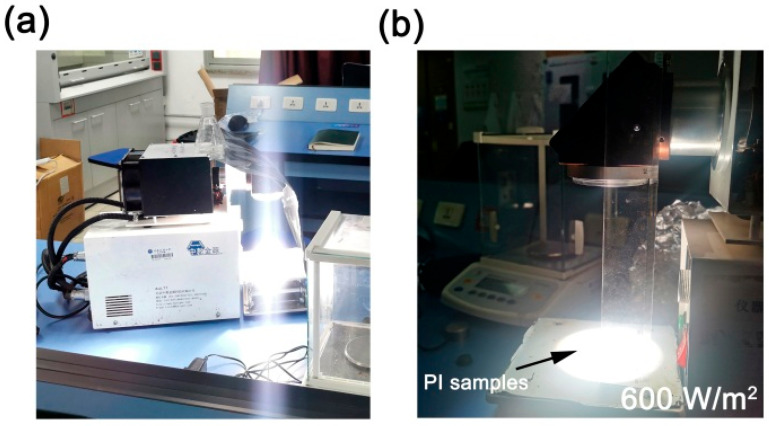
Xenon lamp exposure system. (**a**) test system; (**b**) light source.

The UV irradiation test was performed with a Xenon lamp system (Model: CEL-HXF300, Beijing China Education AuLight Technology Co., Ltd., Beijing, China) with the conditions shown in Figure 2. The distance between the UV-LED array source and the CPI films was controlled to be 7.0 ± 0.5 cm in order to avoid the heat impact of the UV source on the CPI films. The UV exposure time was set to be 1–6 h in the wavelength of 300–2500 nm at 600 W/m^2^.

### 2.3. Preparation of the CPI-0 (HPMDA-DMBZ) Film

Into a 500 mL three-necked, round-bottomed flask equipped with a mechanical stirrer, a Dean–Stark trap and a nitrogen inlet were added DMBZ (21.2290 g, 100 mmol) and the ultra-dry GBL (120 g). The mixture was stirred at room temperature under nitrogen for 30 min to afford a clear diamine solution. Then, HPMDA (22.4170 g, 100 mmol) was added together with an additional GBL (10.9 g). The total solid content of the reaction mixture was 25 wt%. Isoquinoline (0.5 g) and toluene (200 mL) were then added into the reaction system. The reaction mixture was heated to 130–140 °C and the toluene–water azeotrope was distilled out of the system via the Dean–Stark trap. The dehydration process was maintained until no water was detected in the trap (~6 h). Then, the polymerization system was further heated to 180 °C and maintained for another 3 h. After cooling to room temperature, the viscous solution, pale-yellow in color, was poured into an excess of aqueous ethanol (75 vol%) solution to precipitate the flexible and tough white resin. The obtained CPI-0 resin was collected and dried at 80 °C in vacuo for 24 h. Yield: 38.4 g (96%). *M*_n_: 1.09 × 10^5^ g/mol; *M*_w_: 1.99 × 10^5^ g/mol; polydispersity index (PDI): 1.83. ^1^H-NMR (DMSO-*d*_6_, ppm): 7.29–7.11 (*m*, 6H), 3.26 (*m*, 4H), 2.06–2.02 (*m*, 4H), and 1.99 (*m*, 6H).

The dried CPI-0 resin was dissolved into the ultra-dry DMAc (water content <50 ppm) with a solid content of 25 wt%. After stirring for 3 h at room temperature, a homogeneous and viscous CPI-0 solution was obtained. The solution was then filtered through a 0.45 μm Teflon syringe filter. The purified CPI-0 solution was spin-coated on a clean 4-inch (diameter: ~10.2 cm) quartz substrate. The thickness of the CPI films for various measurements was controlled by regulating the spinning rate. Free-standing CPI-0 films with the thicknesses ranged from 10–100 μm were obtained by thermally baking the CPI-0 solution in a nitrogen-purged oven according to the heating procedure of 80 °C/2 h, 150 °C/1 h, 180 °C/1 h, 200 °C/1 h, 250 °C/1 h, and 280 °C/1 h.

### 2.4. Preparation of CPI/HALS Nanocomposite Films

A series of HALS-containing CPI films combined with different loading content of HALS additives (0, 0.1 wt%, 0.3 wt%, 0.5 wt%, and 1.0 wt% based on the CPI-0 matrix) were prepared. Table 1 shows the formulas for the preparation of the CPI/HALS nanocomposite films. The total solid content for each system containing the CPI-0 and the HALS was set to be 15 wt%. CPI-0 and HALS were separately dissolved in DMAc. Then, the obtained HALS/DMAc solution was mixed with the CPI-0/DMAc to afford the final composite solutions for film fabrication. Table 2 further lists the abbreviation and description of the CPI samples in the current work. For convenience, the name of the HALS compounds was abbreviated as “783” for Tinuvin^®^ 783, “791” for Tinuvin^®^ 791, “944” for Chimassorb^®^ 944, and “2020” for Chimassorb^®^ 2020. Four series of CPI/HALS composite films based on CPI-0 matrix and different HALS were named as “CPI-A-X” for the systems containing 2020, “CPI-B-X” for the systems containing 944, “CPI-C-X” for the systems containing 783, and “CPI-D-X” for the systems containing 791, respectively. In the abbreviations, “X” represents the weight ratio of HALS to CPI-0. For convenience, X = 1 stands for HALS/CPI-0 = 0.1 wt%, X = 3 for HALS/CPI-0 = 0.3 wt%, X = 5 for HALS/CPI-0 = 0.5 wt%, and X = 10 for HALS/CPI-0 = 1.0 wt%, respectively.

The detailed synthesis procedure could be illustrated by the preparation of CPI-D-1. First, CPI-0 (3.9475 g) and DMAc (21.0525 g) were added into a 100-mL three-necked flask equipped with a mechanical stirrer. The mixture was stirred at room temperature for 10 h to afford a light-colored viscous solution, to which was added a Tinuvin^®^ 791 (3.9475 mg) solution in DMAc (1.3390 g). The reaction mixture was stirred at room temperature for another 14 h. Then, the obtained homogeneous CPI/HALS solution was purified with the similar procedure reported in Section 2.3. The other series of CPI/HALS solutions were prepared according to the similar procedures mentioned above. The detailed formulas for the preparation of the CPI/HALS composite solutions were listed in Table 1.

CPI-D-1 films were prepared according to the similar procedure reported in Section 2.3. The other series of CPI/HALS composite films were prepared according to the similar procedures mentioned above.

## 3. Results and Discussion

### 3.1. HALS Evaluation

The solubility and thermal stability of four commercially available HALS were separately evaluated. As mentioned before, the ideal HALS for the improvement of the photo-stability of the CPI films should possess good solubility in the good solvents for the CPI resin so as to achieve good miscibility and compatibility between the matrix and the filler. On the other hand, the HALS compounds should have enough thermal stability in order to maintain the structural integrity during the nanocomposite films fabrication at elevated temperatures as high as 280 °C.

**Figure 3:**
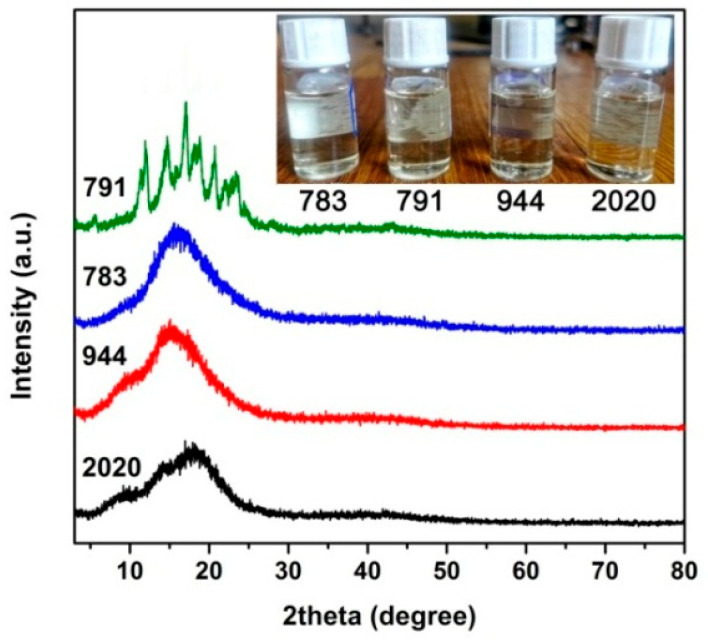
XRD patterns of hindered amine light sensitizer (HALS) compounds. (Insert: solubility of HALS in DMAc at a solid content of 10 wt%).

First, the solubility of the HALS in DMAc was evaluated, and the results are shown in Figure 3, together with the XRD patterns of the HALS. It could be clearly seen that the HALS could be totally dissolved into DMAc at the solid contents of 16.7 wt%. The good solubility of the current HALS in DMAc was mainly attributed to the amorphous or low degree of crystallinity in the compounds, as evidenced by the XRD measurements. All the HALS except 791 exhibited amorphous molecular structures due to the oligomeric nature of the compounds. Table 1 shows the FWHM values of the HALS compounds. It could be clearly seen that the 791 compounds exhibited the lowest FWHM value, indicating the highest crystallinity in the HALS. The crystalline absorptions for 791 in the scattering angles between 10–30° were mainly ascribed to the small molecules of Tinuvin^®^ 770 (*M*_n_ = 480.72 g/mol) components in the HALS (Figure 1). Nevertheless, the other components of 944 oligomers in 791 HALS decreased the total crystallinity of the mixture. The good solubility of the current HALS in DMAc is quite beneficial for their applications in the preparation of CPI/HALS composite films because the highly homogeneous blending with little aggregation in HALS might be achieved for the derived composite films. The intrinsic optical transparency of the pristine CPI-0 film might be maintained.

**Figure 4:**
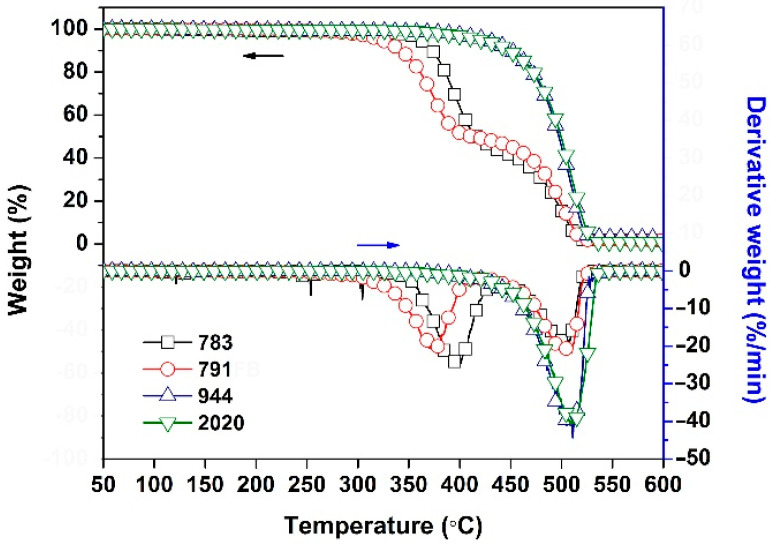
TGA and DTG plots of hindered amine light sensitizers (HALS).

Secondly, the thermal stability of the HALS was investigated by TGA measurements and the results are shown in Figure 4 and Table 3. The HALS showed the good thermal stability before 300 °C and the 5% weight loss temperatures (*T*_5%_) of the HALS increased with the order of 791 < 783 < 2020 < 944. For example, 791 exhibited a *T*_5%_ value of 322.4 °C, which was more than 100 °C lower than that of 944 (*T*_5%_ = 425.5 °C). The inferior thermal stability of 791 and 783 HALS might be due to the small-molecule components in the compounds (Figure 1). This could also be reflected by the derivative TG (DTG) plots of the HALS. According to the DTG plots, 791 and 783 exhibited obvious two-stage decomposition during the TGA measurements. The first thermal decomposition occurred around 370–400 °C, which could be ascribed to the decomposition of the small molecules of Tinuvin^®^ 770 for 791 and Tinuvin^®^ 622 for 783, respectively. The second one was observed in the range of 500–505 °C, which was due to the decomposition of the main components for the HALS. By contrast, the 944 and 2020 compounds with much higher molecular weights showed only one-stage thermal decomposition behaviors. Accordingly, 944 and 2020 exhibited much higher residual weight ratios at elevated temperatures. For example, the 2020 HALS showed the residual weight ratio of 48.7 wt% at 500 °C, which was much higher than those of 783 (15.3 wt%) and 791 (18.2 wt%). Although the current HALS compounds showed different thermal stability, the high initial thermal decomposition temperatures over 320 °C made them good candidates for the photo-stabilization applications for CPI films.

### 3.2. CPI-0 and CPI/HALS Nanocomposite Film Preparation

**Figure 5:**
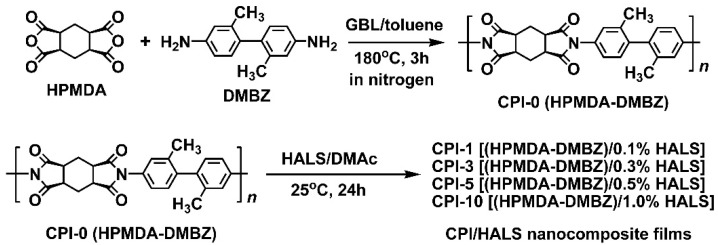
Preparation of CPI-0 and the CPI/HALS nanocomposite films.

A semi-alicyclic CPI-0 (HPMDA-DMBZ) matrix film was prepared according to the one-step high-temperature polycondensation procedure shown in Figure 5. The derived CPI-0 resin was easily soluble in polar aprotic solvents, such as NMP, DMAc, and DMF. In addition, it was also soluble in cyclopentanone (CPA) at room temperature. The good solubility of the resin was mainly due to the less-conjugated molecular structure in the polymer. The CPI-0 resin had the *M*_n_ value of 1.09 × 10^5^ g/mol, the *M*_w_ value of 1.99 × 10^5^ g/mol, and polydispersity index (PDI) of 1.83, indicating the high reactivity during the polymerization. Flexible, tough, and colorless CPI-0 films were prepared by thermally baking the CPI-0 solution at a relatively low temperature of 280 °C.

**Figure 6:**
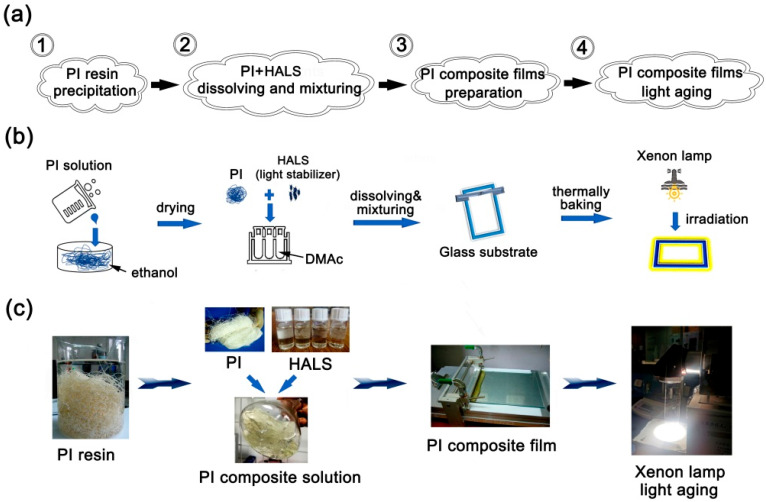
Experimental design and property evaluation of the CPI films. (**a**) flowchart; (**b**) vectorgraph; (**c**) experimental diagram.

Then, based on the CPI-0 matrix, four series, 16 CPI/HALS composite films with different types of HALS and various loading amounts were prepared in total, respectively, according to the procedures shown in Figure 6. The composite solutions were first prepared by blending the CPI-0/DMAc solution and the HALS/DMAc solution with the total solid contents of 15 wt%. Then, the CPI/HALS composite films were fabricated by the similar procedure with the pristine CPI-0 film. Subsequently, the thermal, optical, and UV irradiation investigation of the CPI-0 and CPI/HALS composite films were carried out.

**Figure 7:**
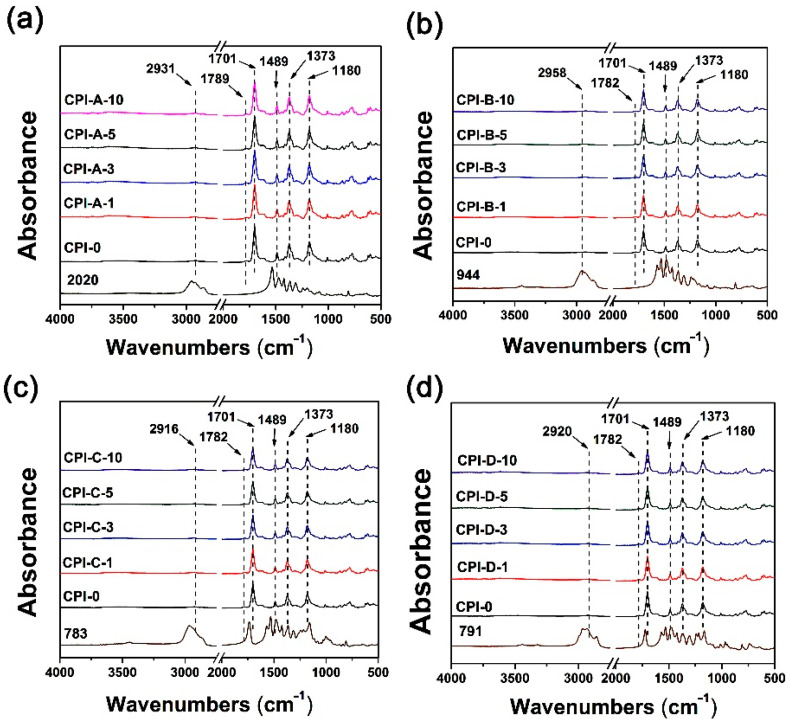
FTIR spectra of CPI/HALS nanocomposite films. (**a**) CPI-A; (**b**) CPI-B; (**c**) CPI-C; (**d**) CPI-D.

First, the chemical structures of the CPI films, together with the corresponding HALS, were identified by the FT-IR measurements and the spectra are shown in Figure 7. It could be clearly observed that all the CPI films demonstrated the similar spectra due to the similar structural compositions. The characteristic absorptions of imide groups, including the asymmetrical stretching vibrations of C=O in imides at 1782–1789 cm^−1^, the symmetrical stretching vibrations of C=O in imides at 1701 cm^−1^, and the stretching vibration of C-N in imides at 1373 cm^−1^ were all observed [31]. Meanwhile, the characteristics absorptions of saturated C-H stretching vibrations at 2916–2958 cm^−1^ assigned to the –CH_3_ groups in the DMBZ units in CPI-0 or the –CH_3_ and –CH_2_– groups in HALS were also detected. The stretching vibrations at 1489 cm^−1^ ascribed to the C=C bonds in the phenyl units in CPI-0 components were detected. In addition, the characteristic absorptions of the HALS were basically overlapped by the absorptions of the CPI-0 due to the very limited amounts of the compounds.

**Figure 8:**
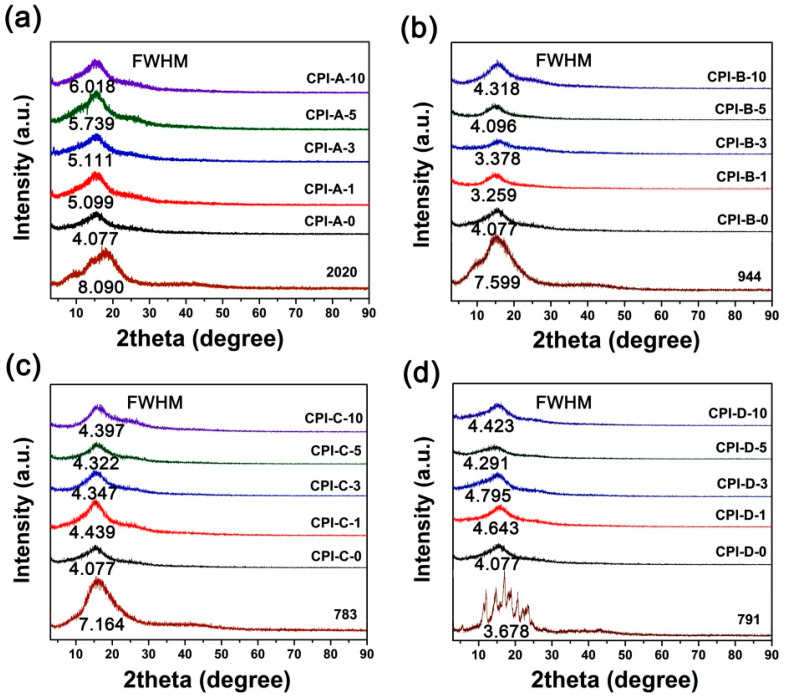
XRD plots of CPI/HALS nanocomposite films. (**a**) CPI-A; (**b**) CPI-B; (**c**) CPI-C; (**d**) CPI-D.

Figure 8 shows the XRD patterns of the CPI films and the corresponding HALS, together with the corresponding FWHM values of the samples. The HALS of oligomeric 2020, 944, and 783 exhibited the amorphous nature, while the 791 containing the small molecules of Tinuvin^®^ 770 (*M*_n_ = 480.72 g/mol) showed the crystalline feature with the scattering angles in the range of 10° to 30°. Nevertheless, all the CPI/HALS exhibited amorphous natures, indicating the successful incorporation of the HALS additives into the pristine CPI-0 matrix. All the CPI/HALS nanocomposite films showed the same level FWHM values with the pristine CPI-0 matrix, indicating that incorporation of HALS at the current loading amounts did not affect the degree of crystallinity for the CPI films. The amorphous nature for the current composite films is beneficial for maintaining the intrinsic optical transparency of the films.

### 3.3. Optical Properties

**Figure 9:**
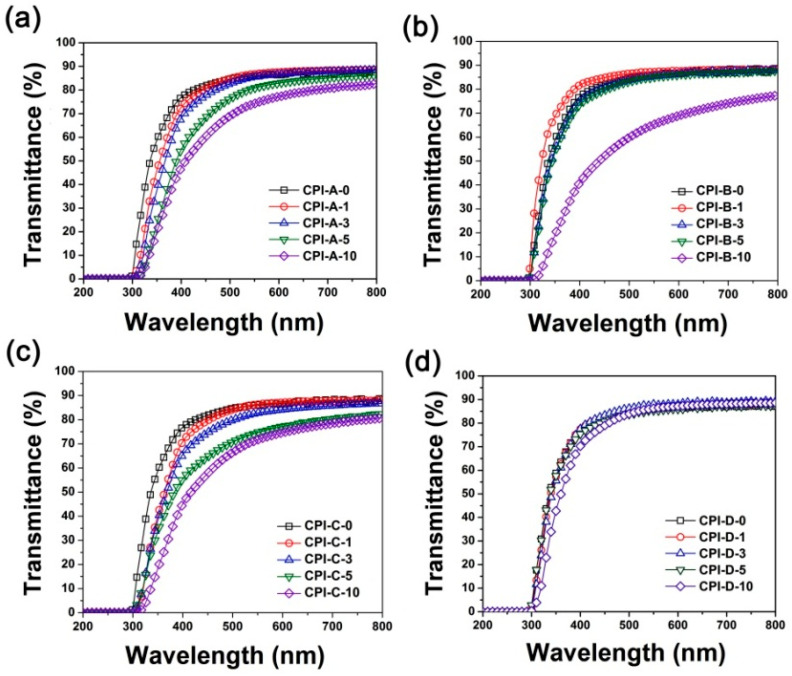
UV-Vis spectra of CPI/HALS nanocomposite films. (**a**) CPI-A; (**b**) CPI-B; (**c**) CPI-C; (**d**) CPI-D.

Influences of the incorporation of different types of HALS with different loading amounts on the optical properties of the CPI/HALS composite films were quantitatively investigated by comparing the optical transmittance of the composite films with the pristine CPI-0 matrix. Figure 9 shows the UV-Vis spectra of four series of CPI/HALS composite films and the optical data are listed in Table 4.

**Figure 10:**
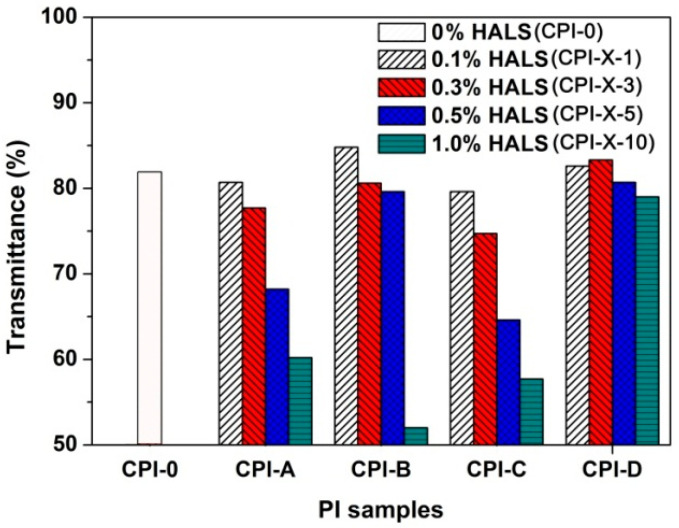
Comparison of optical transmittances of the CPI-0 and the CPI/HALS nanocomposite films at the wavelength of 450 nm.

Basically, the optical transmittances of the pristine CPI-0 films deteriorated to some extent with the incorporation of HALS. It demonstrates the negative effects of the HALS compounds on the optical properties of the CPI films, which might be due to the large amounts of secondary amine and tertiary amine groups in the HALS. Among the CPI/HALS composite films, the optical transmittance of CPI-D systems decreased the least due to the incorporation of HALS. That is to say, the introduction of 791 type of HALS did not have a significant effect on the optical transmittances of the composite films. Furthermore, incorporation of the other three types of oligomeric HALS into the CPI-0 film apparently reduced the optical transmittance of the composite films, although the cutoff wavelength (*λ*_cut_) of the CPI films changed only a little. For example, at the same loading amounts of 10 wt%, the four series of the CPI composite films showed the decreased optical transmittances at the wavelength of 450 nm (*T*_450_) with the order of CPI-D-10 (79.0%) > CPI-C-10 (57.7%) > CPI-A-10 (60.2%) > CPI-B-10 (52.0%) as compared with the pristine CPI-0 matrix (*T*_450_ = 81.9%). It is the same trend for optical transmittances at the wavelength of 400 nm (*T*_400_) of the CPI films. Figure 10 illustrates the effects of the incorporation of different types of HALS on the *T*_450_ values of the composite films. The better optical properties of the CPI-D films might be due to the fact that 791 HALS, as a mixture of small-molecule Tinuvin^®^ 770 and the oligomeric 944, has a better dispersion and distribution in the CPI-0 matrix than those of the other oligomeric HALS compounds. In view of the better optical properties of the CPI-D system, they were used for the following UV irradiation tests.

### 3.4. Photo-Degradation Behaviors of the CPI-D Nanocomposite Films

**Figure 11:**
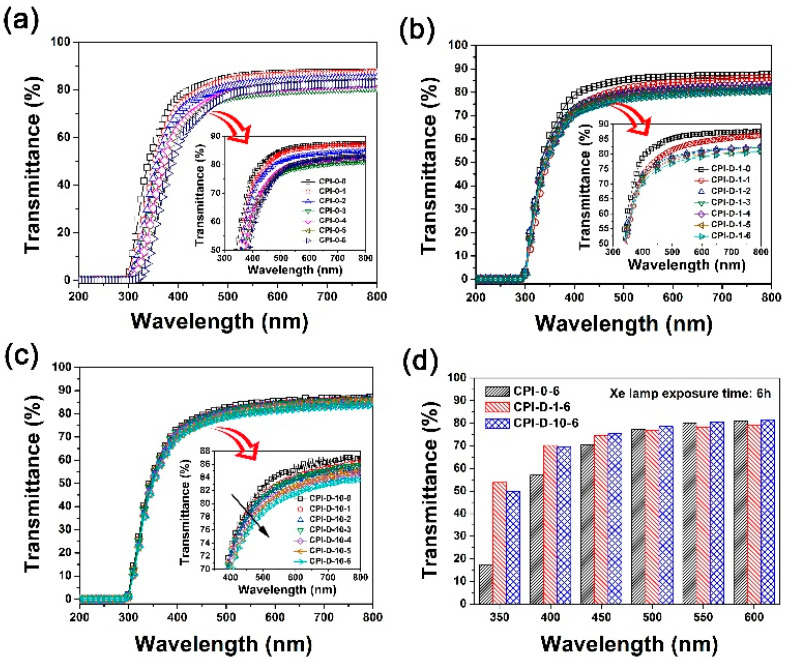
UV-Vis spectra of CPI-0 film and CPI-D nanocomposite films after Xenon lamp exposure. (**a**) CPI-0; (**b**) CPI-D-1 (0.1% 791); (**c**) CPI-D-10 (1.0% 791); (**d**) comparison of optical transmittances of the films after UV exposure for 6 h.

**Figure 12:**
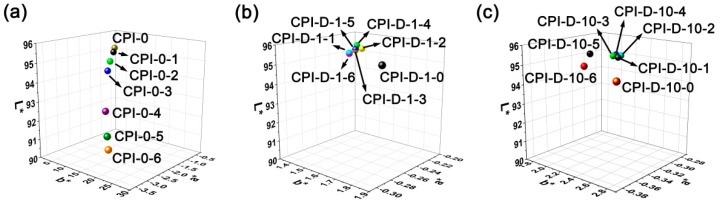
Three-dimensional (3D) CIE Lab parameters of CPI-0 and CPI-D nanocomposite films after Xenon lamp exposure. (**a**) CPI-0; (**b**) CPI-D-1; (**c**) CPI-D-10.

The photo-degradation behaviors of the CPI-0 and the CPI-D nanocomposite films were comparatively investigated by the UV-Vis and the CIE Lab color parameters’ measurements. According to the abbreviations of the CPI samples listed in Table 2, the CPI-0-X and CPI-D-X films irradiated by the UV sources for Y hours were named as “CPI-0-X-Y” and “CPI-D-X-Y”, respectively. Figure 11 depicts the changing of the optical transmittances of the representative CPI films, including CPI-0 (Figure 11a), CPI-D-1 (Figure 11b), and CPI-D-10 (Figure 11c) with the increasing of the UV irradiation time. Figure 12 compares the three-dimensional (3D) CIE Lab parameters of CPI-0 and CPI-D films after Xenon lamp exposure. The optical data before and after UV irradiation for specific time are shown in Table 5.

Both of the optical transmittances and the CIE color parameters deteriorated with the UV Xenon lamp exposure. For the pristine CPI-0 system, the transmittance at the wavelength of 350 nm (*T*_350_) values decreased from the initial 55.7% (CPI-0-0) to the final 17.5% (CPI-0-6) after exposure for 6 h (Figure 11a). The CLE Lab color parameters showed the changes of L* from 95.12 to 91.38, yellow indices (b*) from 3.38 to 21.95, and haze from 1.46% to 9.33%, respectively. Thus, it demonstrates that the CPI-0 (HPMDA-DMBZ) film was intrinsically UV sensitive and the film mainly darkened (L*↓) and became yellow (b*↑) and opaque (haze↑) after UV irradiation. This is consistent with the photo-oxidation phenomena caused by UV irradiation. Therefore, it could be expected that the UV irradiation resistance of the CPI-0 film can be significantly improved with the incorporation of the HALS.

It could be seen from Figure 11b,c that the CPI-D-1 and CPI-D-10 films showed the initial *T*_350_ values of 61.4% and 54.3%, respectively. After UV irradiation for 1–6 h, the CPI-D-1 films exhibited the *T*_350_ values in the range of 53.4–55.9%, which were 87–91% of the initial values, while the CPI-D-10 films showed the higher *T*_350_ value retentions of 92–98%. For example, the CPI-D-1-6 and CPI-D-10-6 films showed the *T*_350_ values of 53.8% and 53.4%, respectively, which were 87.6% and 98.3% of the pristine films. Figure 11d summarizes and compares the transmittance–wavelength relationships of the CPI films after 6 h of UV irradiation, including CPI-0-6, CPI-D-1-6, and CPI-D-10-6. It could be concluded that incorporation of the 791 HALS could significantly improve the anti-UV irradiation stability of the CPI-0 films. Secondly, the addition of 1 wt% 791 could better improve the UV irradiation resistance of the CPI films than those of the systems containing 0.1 wt% of the HALS. However, the former was suffered from the deterioration of the optical transmittances to some extent.

### 3.5. Thermal Properties of the CPI-D Nanocomposite Films

**Figure 13:**
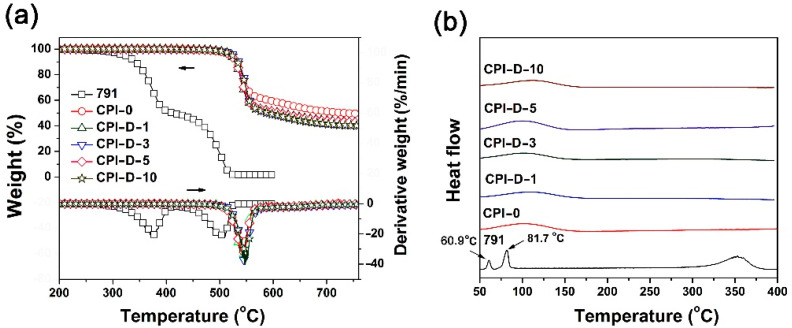
Thermal properties of CPI-0 film and CPI/791 nanocomposite films under nitrogen flow. (**a**) TGA and DTG; (**b**) DSC.

Effects of the incorporation of 791 HALS on the thermal stability of the CPI composite films were studied by TGA, DSC, and TMA measurements, respectively, and the data are listed in Table 6. Incorporation of 791 HALS maintained the thermal stability of the pristine CPI-0 film according to the TGA plots shown in Figure 13a. For example, the CPI-D-10 film showed the 5% weight loss temperature (*T*_5%_) of 524.1 °C in nitrogen, which was close to that of the CPI-0 matrix (*T*_5%_ = 521.5 °C). Although 791 showed apparent two-stage decomposition behaviors according to the derivative TGA (DTG) plots, all the CPI films exhibited only one-stage decomposition with the maximum decomposition speed temperatures (*T*_max_) around 540–550 °C. The residual weight ratios at 750 °C (*R*_w750_) of the CPI composite films were in the range of 40.3–42.9 wt%, which were slightly lower than that of the pristine CPI-0 matrix (*R*_w750_ = 51.2%). It is mainly due to the thermally unstable nature of the aliphatic chains in 791 HALS at elevated temperatures. All of the CPI composite films did not exhibit detectable glass transition behaviors in the DSC measurements (Figure 13b) in the temperature range of 50–400 °C, although the 791 HALS showed two melting absorptions at 60.9 °C and 81.7 °C, respectively.

**Figure 14:**
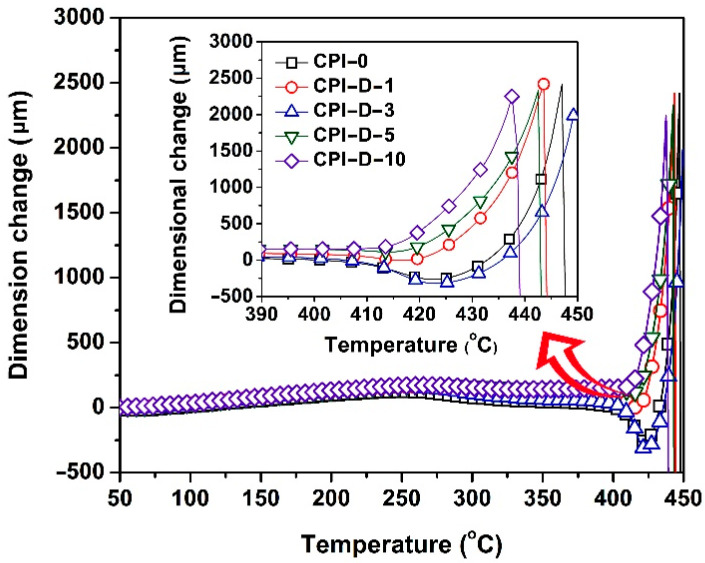
TMA of CPI-0 film and CPI/791 nanocomposite films under nitrogen flow.

Figure 14 shows the TMA curves of the CPI films. Incorporation of the 791 HALS slightly deteriorated the high-temperature dimensional stability of the pristine CPI-0 film. For example, the CPI-D-10 film showed a CTE value of 55.9 × 10^−6^/K in the temperature range of 50–250 °C, which was higher than that of CPI-0 matrix (CTE = 46.9 × 10^−6^/K). The plasticization effects of the small-molecule Tinuvin^®^ 770 and the oligomeric 944 in 791 HALS might increase the CTE values of the composite films.

## 4. Conclusions

A series of semi-alicyclic CPI composite films with enhanced UV-stability were designed and developed via the blending methodology of the CPI film matrix with the HALS types of photo-stabilizers. The selected 791 HALS showed good thermal stability and good miscibility with the CPI-0 matrix. Incorporation of the 791 HALS could significantly improve the UV irradiation stability of the CPI-0 film at the loading amounts of 0.1 wt% without obviously scarifying the optical transparency and thermal stabilities of the composite films. The current research provided an efficient pathway for developing high-performance CPI films for advanced optoelectronic applications in which long-term UV irradiation has to be concerned.

## Figures and Tables

**Table 1:** Formulas for the preparation of CPI/HALS nanocomposite films.

CPI	CPI-0, DMAc (g, g)	HALS, DMAc (mg, g)	*M*_HALS_ */M*_CPI_
CPI-1	3.9475, 21.0525	3.9475, 1.3390	0.1%
CPI-3	3.9475, 21.0525	11.8425, 1.3840	0.3%
CPI-5	3.9475, 21.0525	19.7375, 1.4285	0.5%
CPI-10	3.9475, 21.0525	39.4750, 1.5405	1.0%

**Table 2:** Abbreviation and description of the CPI samples in the current work.

Samples	Description
2020	Chimassorb^®^ 2020 (BASF, Ludwigshafen, Germany)
944	Chimassorb^®^ 944 (BASF, Ludwigshafen, Germany)
783	Tinuvin^®^ 783 (BASF, Ludwigshafen, Germany)
791	Tinuvin^®^ 791 (BASF, Ludwigshafen, Germany)
CPI-0	CPI (HPMDA-DMBZ) matrix
CPI-1	CPI/HALS nanocomposite film with the *M*_HALS_/*M*_CPI_ = 0.1%
CPI-3	CPI/HALS nanocomposite film with the *M*_HALS_/*M*_CPI_ = 0.3%
CPI-5	CPI/HALS nanocomposite film with the *M*_HALS_/*M*_CPI_ = 0.5%
CPI-10	CPI/HALS nanocomposite film with the *M*_HALS_/*M*_CPI_ = 1.0%
CPI-A-X	Series A, CPI-0/2020 composite films; X: contents of 2020 (X = 1, 0.1%; X = 3, 0.3%; X = 5, 0.5%; X = 10, 1.0%)
CPI-B-X	Series B, CPI-0/944 composite films; X: contents of 944 (X = 1, 0.1%; X = 3, 0.3%; X = 5, 0.5%; X = 10, 1.0%)
CPI-C-X	Series C, CPI-0/783 composite films; X: contents of 783 (X = 1, 0.1%; X = 3, 0.3%; X = 5, 0.5%; X = 10, 1.0%)
CPI-D-X	Series D, CPI-0/791 composite films; X: contents of 791 (X = 1, 0.1%; X = 3, 0.3%; X = 5, 0.5%; X = 10, 1.0%)
CPI-D-X-Y	CPI-0/791 composite film with different UV exposure time; X: contents of 791 (X = 1, 0.1%; X = 10, 1.0%); Y: Xenon lamp exposure time (Y = 1, 1 h; Y = 1.5, 1.5 h; Y = 2, 2 h; Y = 3, 3 h; Y = 4, 4 h; Y = 5, 5 h; Y = 6, 6 h)

**Table 3:** Thermal stability of the HALS compounds.

HALS	FWHM ^1^ (°)	*T*_5%_ ^2^ (°C)	*T*_10%_ ^2^ (°C)	*T*_max1_ ^2^ (°C)	*T*_max2_ ^2^ (°C)	*R*_w500_ ^2^ (%)
783	7.164	361.9	372.8	399.7	502.3	15.3
791	3.678	322.4	342.3	376.3	503.7	18.2
944	7.599	425.5	450.3	NA ^3^	510.8	45.2
2020	8.090	420.5	448.8	NA	509.0	48.7

^1^ Full-width at half-maximum. ^2^ *T*_5%_, *T*_10%_: 5% and 10% weight loss temperature, respectively; *T*_max_: the temperature at the maximum decomposition speed; *R*_w500_: residual weight ratio at 500 °C in nitrogen. ^3^ Not applicable.

**Table 4:** Optical properties of CPI/HALS nanocomposite films.

Property	CPI-0	Series A (2020)				Series B (944)				Series C (783)				Series D (791)			
		A-1	A-3	A-5	A-10	B-1	B-3	B-5	B-10	C-1	C-3	C-5	C-10	D-1	D-3	D-5	D-10
*λ*_cut_ ^1^ (nm)	292	290	293	298	293	289	293	290	294	297	292	294	300	293	294	292	294
*T*_400_ ^2^ (%)	76.9	72.7	68.1	54.8	47.0	81.7	74.8	73.5	40.9	70.9	65.6	55.5	45.1	77.5	77.5	75.3	70.2
*T*_450_ ^2^ (%)	81.9	80.7	77.7	68.2	60.2	84.8	80.6	79.6	52.0	79.6	74.7	64.6	57.7	82.6	83.3	80.7	79.0

^1^ Cutoff wavelength. ^2^ *T*_4__00_, *T*_450_: Transmittance at 400 nm and 450 nm at a thickness of 25 μm, respectively.

**Table 5:** Optical properties of CPI/791 nanocomposite films after Xenon lamp exposure for 6 h.

CPI Samples	*T*_350, Xe_ ^1^ (%)	L*_Xe_ ^2^	b*_Xe_ ^2^	haze_Xe_ ^2^ (%)
CPI-0-0	55.7	95.12	3.38	1.46
CPI-0-1	45.4	94.92	3.82	3.12
CPI-0-2	42.7	94.55	5.65	4.97
CPI-0-3	33.7	94.23	8.22	7.03
CPI-0-4	30.2	92.71	15.59	7.12
CPI-0-5	21.2	91.91	20.60	8.81
CPI-0-6	17.5	91.38	21.95	9.33
CPI-D-1-0	61.4	95.46	1.84	0.69
CPI-D-1-1	53.8	95.38	1.51	3.46
CPI-D-1-2	55.0	95.34	1.46	3.19
CPI-D-1-3	53.4	95.33	1.46	3.87
CPI-D-1-4	55.6	95.36	1.38	4.20
CPI-D-1-5	55.9	95.37	1.46	3.10
CPI-D-1-6	53.8	95.36	1.51	3.34
CPI-D-10-0	54.3	94.96	2.82	3.38
CPI-D-10-1	53.4	94.93	2.11	5.33
CPI-D-10-2	50.2	94.93	2.07	5.95
CPI-D-10-3	53.1	94.97	2.04	5.57
CPI-D-10-4	50.8	94.95	2.00	5.53
CPI-D-10-5	51.8	95.25	1.97	5.61
CPI-D-10-6	49.9	94.95	2.16	7.63

^1^ *T*_350, Xe_: Transmittance at the wavelength of 350 nm at a thickness of 25 μm after Xenon lamp exposure; ^2^ L*_Xe_, b*_Xe_, haze*_Xe_*: Lightness, yellow indices, and haze for CPI films after Xenon lamp exposure.

**Table 6:** Thermal properties of the CPI-0 film and CPI/791 nanocomposite films.

PI Samples	*T*_5%_ ^1^ (°C)	*T*_10%_ ^1^ (°C)	*T*_max_ ^1^ (°C)	*R*_w750_ ^1^ (%)	CTE ^1^ (10^−6^/K)
CPI-0	521.5	531.2	541.9	51.2	46.9
CPI-D-1	521.6	528.8	542.7	42.9	53.8
CPI-D-3	526.3	535.0	550.1	41.1	53.1
CPI-D-5	520.4	529.4	544.0	42.7	55.6
CPI-D-10	524.1	533.6	548.1	40.3	55.9

^1^ *T*_5%_, *T*_10%_: 5% and 10% weight loss temperature, respectively; *T*_max_: temperature at which the most rapid decomposition was recorded; *R*_w7__50_: residual weight ratio at 750 °C; CTE: Coefficient of linear thermal expansion recorded between 50–250 °C.

## Data Availability

Data are contained within the article.
